# Proteomic analyses of the regulatory mechanisms underlying *Pochonia chlamydosporia* infection in *Parascaris equorum* eggs

**DOI:** 10.3389/fmicb.2025.1644912

**Published:** 2025-10-15

**Authors:** Yuan Ma, Jinbao Lv, Luyao Hao, Zhengyi Li, Chengyu Ma, Rui Wang, Lili Jiang, Zhaobin Fan

**Affiliations:** ^1^College of Veterinary Medicine, Inner Mongolia Agricultural University, Hohhot, China; ^2^Key Laboratory of Clinical Diagnosis and Treatment of Animal Diseases, Ministry of Agriculture, National Animal Medicine Experimental Teaching Center, Hohhot, China; ^3^National Center of Technology Innovation for Dairy, Hohhot, China; ^4^Zhongnong Dong Jun Animal Diagnosis Technology (Beijing) Co., Ltd., Beijing, China; ^5^College of Pharmacy, Heze University, Heze, China

**Keywords:** *Pochonia chlamydosporia*, 4D-DIA proteomics, DEPs, insecticidal mechanism, qPCR

## Abstract

**Background:**

*Pochonia chlamydosporia* is an important egg-parasitic fungus with potential applications in the biological control of parasitic pests. However, the protein-response mechanisms during *P. chlamydosporia* infection of nematode eggs remain unclear. In this study, we employed four-dimensional data-independent acquisition (4D-DIA) proteomic sequencing to analyze the changes in the mycelial proteome of *P. chlamydosporia* at different infection stages.

**Results:**

In total, 4,293 differentially expressed proteins (DEPs) were identified, which were mainly involved in energy metabolism, protein synthesis and modification, oxidative stress, and other key biological processes. In the early stages of infestation, the fungus rapidly adapted to the host environment by enhancing metabolism and protein synthesis, initiating the infestation mechanism, and simultaneously enhancing its antioxidant capacity to cope with the host defense response. At later stages, it fine-tuned the metabolic pathways and enhanced DNA replication to maintain proliferation and continuously strengthened the antioxidant response to host oxidative stress. In addition, the number of proteins related to fungal transporter activity varied significantly after induction, indicating that a variety of transmembrane proteins may be involved in host recognition, adhesion, and formation of invasive structures.

**Conclusion:**

This study provides critical insights into the molecular mechanisms underlying *P. chlamydosporia* parasitic activity and establishes a theoretical foundation for the development of novel biocontrol strategies for this fungus.

## Introduction

*Pochonia chlamydosporia* is an important opportunistic nematophagous fungus and a representative species of egg-parasitic fungi. *Pochonia chlamydosporia* is widely used as a biocontrol agent. This fungus is distributed worldwide and may adopt saprotrophic and endophytic lifestyles (Li et al., 2022). It represents a major class of fungal biocontrol agents that specifically target the eggs of parasitic nematodes, which is a critical life stage for controlling these pests (Mukhtar et al., 2013; Saeed et al., 2023). Clinical trials have confirmed that *P. chlamydosporia* can be employed both *in vitro* and *in vivo* for effective management of animal parasites. Crucially, its safety profile is well-established, posing no harm to animals or humans, which is essential for practical biocontrol applications (Araujo et al., 2013; García et al., 2004a, 2004b, 2008; Ma et al., 2025). Several studies have demonstrated the efficacy of *P. chlamydosporia* in reducing gastrointestinal nematode infections in horses, cattle, and sheep (Braga et al., 2010, 2012; Carvalho et al., 2010; de Carvalho et al., 2013; Dias et al., 2013; Ferreira et al., 2011; Oliveira et al., 2021; Thapa et al., 2018; Vieira et al., 2020; Araujo et al., 2013).

At present, the mechanism by which *P. chlamydosporia* identifies nematodes and eggs is not clear; however, the known virulence factors mainly include chitinases and serine proteases. Upon contact with nematode eggs, the fungus produces adhesive structures and specialized infection pegs at the hyphal tips (García et al., 2004b; Zhu, 2017; Nie, 2019). Adhesion is followed by the secretion of a battery of lytic enzymes, including proteases, chitinases, and lipases (Braga et al., 2010). These enzymes degrade eggshell components (Esteves et al., 2009), enabling mechanical penetration by the infection pegs and subsequent hyphal invasion. Once inside, the fungus utilizes the egg contents for growth, leading to characteristic crumpling, deformation, and destruction of the embryo, and, ultimately, egg death and disintegration, completing the parasitism (García et al., 2004b). Genomic and transcriptomic analyses of *P. chlamydosporia* indicate that genes upregulated during parasitism are involved in diverse functions such as metabolism, cell signaling, transport, gene regulation, and DNA repair (Manzanilla-López et al., 2009; Rosso et al., 2011; Shen et al., 2015).

Despite the substantial advances in our understanding of the genetics and basic parasitism processes of *P. chlamydosporia*, a critical knowledge gap persists at the functional proteome level. While genomic and transcriptomic studies predict potential gene functions, they do not directly reveal the identity, abundance, post-translational modifications, interactions, or *in situ* activity of the key effector proteins (e.g., specific enzyme isoforms, adhesion molecules, and signaling proteins) responsible for the critical steps of adhesion, penetration, and nutrient acquisition during egg parasitism. Thus, this study aimed to understand the underlying mechanisms of this egg-parasitic fungus by performing total protein analysis of *P. chlamydosporia* mycorrhizae using a four-dimensional (4D)-data-independent acquisition (DIA) proteomic approach. A comprehensive understanding of this functional proteome is essential to fully elucidate the molecular mechanisms underlying the biocontrol efficacy of *P. chlamydosporia* and guide the rational development of more efficient and stable biocontrol formulations.

## Materials and methods

### Preparation of *Parascaris equorum* eggs

Equine *Parascaris equorum* females were collected, and the eggs of *Parascaris equorum* were collected directly from their uterus and then sterilized using 1% NaClO solution to prepare a suspension of 10 eggs/μL for the subsequent experiments.

### Fungal culture

**Figure 1:**
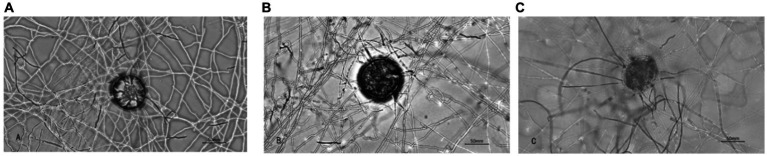
The course of action of *P. chlamydosporia* on the eggs of *Parascaris equorum* [**(A**) 24 h; **(B)** 48 h; **(C)** 96 h].

On the basis of observations of fungal interactions with insect eggs, the time points for sampling in proteomic studies were determined to be 24, 48, and 96 h, corresponding to Groups 1, 2, and 3, respectively. Figure 1 shows the process of infestation, with the three time points corresponding to the pre-infestation, mid-infestation, and post-infestation phases, respectively, of insect eggs by the fungi. *P. chlamydosporia* cultured for 3 d was designated the starting point of the experiment, i.e., the 0 h sample, to which 200 μL of *Parascaris equorum* eggs were added for induction, designated as Group A. A blank control group (Group B) was established by adding 200 μL sterile water instead of the egg suspension. At the corresponding time points, 200 mg of mycelia were collected, dispensed into 1.5-mL centrifuge tubes, and stored in the refrigerator at −80 °C. The samples were stored in the refrigerator at −80 °C. Three biological replicates were used in all the experiments.

### Fungal mycelial protein extraction

Proteins were extracted from tissue samples using SDT lysis buffer [4% sodium dodecyl sulfate (SDS), 100 mM dithiothreitol (DTT), and 100 mM Tris–HCl, pH 8.0]. The samples were boiled for 3 min and then subjected to ultrasonication. The supernatant was collected, and proteins were quantified using a bicinchoninic acid (BCA) protein assay kit.

### Protein assay

For each group of samples, 15 μg of protein samples was obtained, added to 5 × sampling buffer, boiled in a water bath for 5 min, and subjected to 10% SDS-polyacrylamide gel electrophoresis (PAGE).

### Protein digestion

Protein samples were enzymatically digested using the filter-aided sample preparation (FASP) method as follows: DTT was added to each sample to a final concentration of 100 mM, and the samples were heated in a boiling water bath for 5 min and then cooled to room temperature. Two hundred microliters of UA buffer (8 M urea, 150 mM Tris–HCl, pH 8.0) were added and mixed thoroughly, and the solution was transferred to a 10-kDa ultrafiltration centrifuge tube and centrifuged at 12,000 r/min for 15 min. The filtrate was discarded, and the centrifugation procedure was repeated by adding 200 μL of UA buffer again. Subsequently, 100 μL of iodoacetamide (IAA) solution (50 mM IAA dissolved in UA buffer) was added, shaken at 600 r/min for 1 min, incubated for 30 min at room temperature in the dark, and then centrifuged at 12,000 r/min for 10 min. Next, the sample was washed twice with 100 μL of UA buffer and centrifuged at 12,000 r/min for 10 min each time.

For the enzymatic reaction, 40 μL of trypsin buffer (containing 6 μg of trypsin dissolved in 40 μL of NH_4_HCO buffer) was added; the mixture was oscillated at 600 r/min for 1 min and then incubated at 37 °C for 16–18 h. At the end of the reaction, the collection tube was replaced with a new tube; the mixture was centrifuged at 12,000 r/min for 10 min; the filtrate was collected; and the reaction was terminated by adding an appropriate amount of 0.1% trifluoroacetic acid (TFA) solution. The digested peptides were desalted on a C18 column and lyophilized under vacuum. The lyophilized peptides were resolubilized with 0.1% formic acid, and the peptide concentration was determined by liquid chromatography-mass spectrometry (LC–MS).

### DIA analysis of mass spectrometry data

Peptide samples were separated using a Vanquish Neo ultra-high-performance liquid chromatography system (Thermo Scientific). The mobile phases were configured as follows: phase A was an aqueous solution containing 0.1% formic acid and phase B was an acetonitrile–water mixture containing 0.1% formic acid (80% acetonitrile). The column was equilibrated with 96% A phase before use. The sample was first injected into a trap column (PepMap Neo 5 μm C18; inner diameter, 300 μm; length, 5 mm; Thermo Scientific) and then into an analytical column (μPAC Neo high-throughput column; Thermo Scientific) for gradient elution.

The separated peptides were analyzed by DIA using an Orbitrap Astral mass spectrometer (Thermo Scientific). The mass spectrometry data were finally integrated using the DIA-NN software to complete the database retrieval and quantitative analysis of proteins.

### Sequence database searching

DIA MS data were analyzed using DIA-NN 1.8.1. The database used was uniprotkb-Metacordyceps chlamydosporia (Nematophagous fungus) (Pochonia chlamydosporia) [280754]-14275-20241104.fasta, obtained from: https://www.uniprot.org/taxonomy/280754. Trypsin was selected as the digestion enzyme. For the database search, the maximal missed cleavage sites was defined as 1, and the mass tolerance was defined as 10 ppm for precursor ions and 10 ppm for fragment ions. Carbamidomethylation of cysteines was defined as a fixed modification, whereas acetylation of the protein N-terminal and oxidation of methionine were set as variable modifications for database searching. The maximum number of variable modifications was 1. The peptide length range was set to 7–30. The charge of the peptide ranged from 1 to 4. The fragment ion m/z range was 150–2000. The database search results were filtered and exported with a < 1% false discovery rate (FDR) at the peptide-spectrum-matched and protein levels.

### Bioinformatics analysis

Bioinformatics analysis was performed using Microsoft Excel and R statistical computing software. Sequence annotation information was obtained from UniProtKB/SwissProt, Kyoto Encyclopedia of Genes and Genomes (KEGG), and the Gene Ontology (GO) database. To ensure the validity and accuracy of the subsequent raw letters and statistical analyses, in accordance the general principle, in the protein identification form, we first screened the sample experimental data to ensure that at least 50% of the identified proteins corresponding to the sample groups were retained without null-value data, and then filled the data with the remaining null values and performed the statistical analyses, which were performed with the default use of the *t*-test (Student’s *t*-test) combined with the method of fold change (FC, the ratio of the mean value of expression between the two groups). Differentially expressed proteins (DEPs) were screened out by identifying proteins that met the screening criteria of expression difference greater than 1.5-fold (upward and downward adjustments) and a *p* < 0.05, and the DEPs were subjected to GO and KEGG enrichment analysis using Fisher’s exact test with FDR correction for multiple testing. The enriched GO and KEGG pathways were statistically significant at *p* < 0.01 according to Fisher’s exact test.

### Real-time fluorescence quantitative reverse transcription–polymerase chain reaction

Mycelial RNA from different infestation periods was extracted and reverse-transcribed into complementary DNA (cDNA), and the differentially expressed genes (DEGs) were verified by fluorescence quantitative polymerase chain reaction (PCR). Primer 5.0 was used to design the primers for fluorescence quantitative PCR, and 18S rRNA was the internal reference gene. Relative expression was calculated using the 2^-ΔΔCt method^. The data are shown as the means ± standard error of the mean (SEM) of three independent experiments.

## Results

### Screening of *P. chlamydosporia* differential proteins

**Figure 2:**
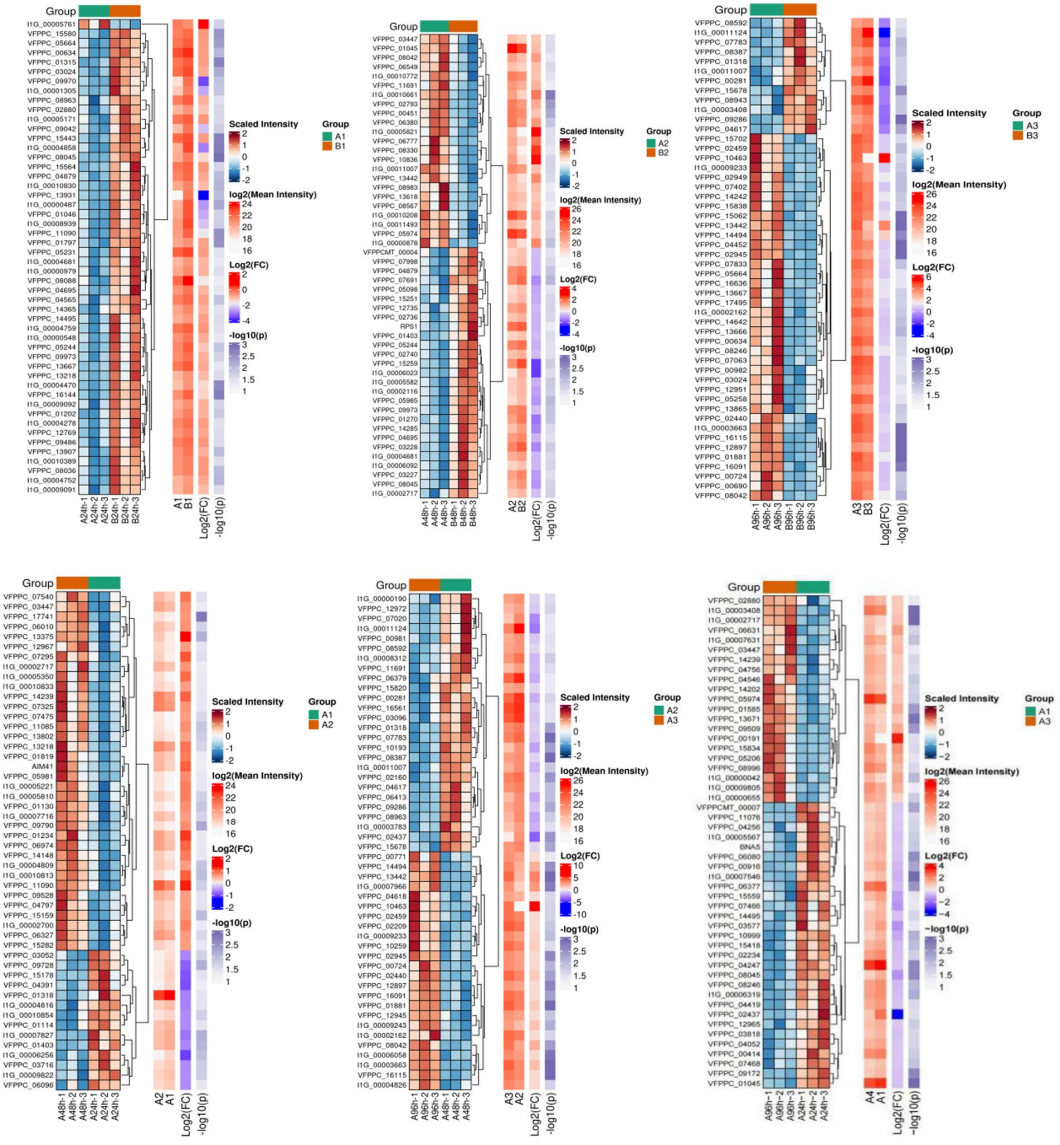
Cluster analysis of differentially expressed proteins.

Proteomic analysis of different infestation periods of *P. chlamydosporia* on the eggs of *Parascaris equorum* was performed using the DIA technique, and the mycelial proteins induced by *Parascaris equorum* (Group A) and sterile water (Group B) at different infestation stages of the egg-parasitic fungus *P. chlamydosporia* were analyzed. The results revealed 8,875 proteins, 108,118 peptides, 106,902 peptides, and 80,742 unique peptides (Table 1 and Figure 2). Proteins that met the screening criteria of an expression difference greater than 1.5-fold (up- and downregulated) and a *p* < 0.05 were regarded as significant DEPs. The number of DEPs in each group is presented in Table 1. Data analysis revealed that 1,597, 245, and 607 proteins showed differential abundance from 0 to 24 h, 24 to 48 h, and 48 to 96 h, respectively. The proteome of the mycelia induced by eggs of *Parascaris equorum* showed 655 DEPs at 24 h in comparison with the findings in the control group (group B) induced by sterile water. Among these proteins, 309 were upregulated and 346 were downregulated. At 48 h, 353 DEPs were identified, of which 196 were upregulated and 157 were downregulated. At 96 h, 790 DEPs were identified, of which 508 were upregulated and 282 were downregulated. These results indicated that protein expression in mycelia induced by *Parascaris equorum* egg extracts differed from that in the control, indirectly suggesting major changes in the physiological activity of the fungus. Analysis of the processes in 24-h (A1/B1), 48-h (A2/B2), and 96-h (A3/B3) samples from both groups revealed that the highest scores were recorded for chitinase, lipase, serine protease, glucanase, pectinase, and cellulase, which are distinct protein hydrolases that may be associated with the process of infestation of the eggs of *Parascaris equorum.* These distinct proteases may contribute to the virulence of the fungus in the pathogens and promote the colonization of the eggs.

**Table 1:** Data for differentially expressed proteins.

Comparison group	Upregulated	Downregulated	Number of DEPs
A1 vs. B1	309	346	655
A2 vs. A1	142	103	245
A2 vs. B2	196	157	353
A3 vs. A2	323	284	607
A3 vs. B3	508	282	790
A1 vs. A3	688	955	1,643

DEP, differentially expressed protein.

### Subcellular localization of whole proteins

**Figure 3:**
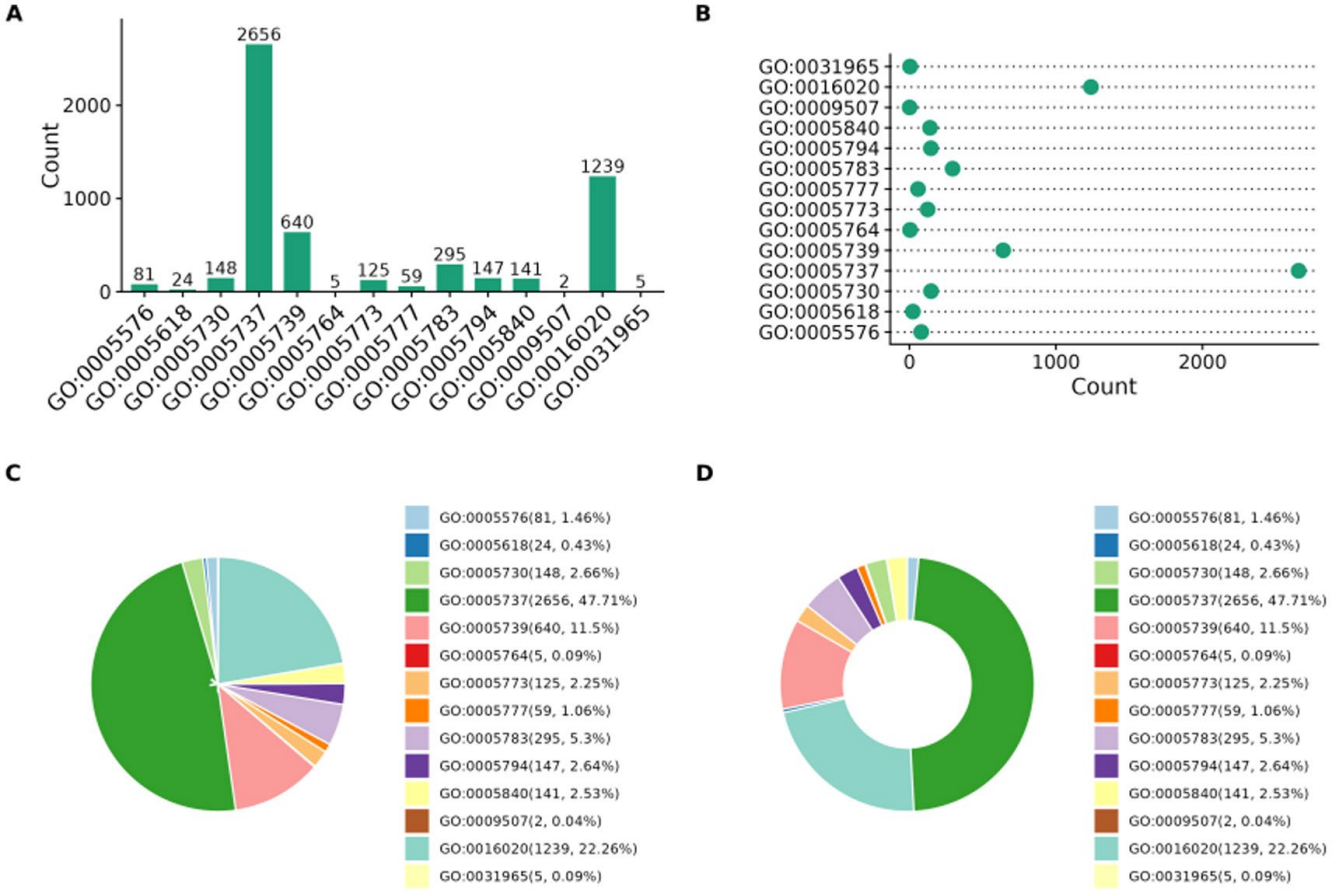
Subcellular localization of whole proteins. **(A)** Bar chart **(B)** Scatter plot **(C)** Pie chart **(D)** Donut chart.

The subcellular localization of whole proteins was annotated and counted by analyzing the cellular component (CC) classification of the GO database. The results showed that the subcellular localization of the whole proteins was mainly focused in the cytoplasm, nuclear membrane, and mitochondria, and the pathway was enriched in GO:005737, as shown in Figure 3.

### GO functional analysis of DEPs

**Figure 4:**
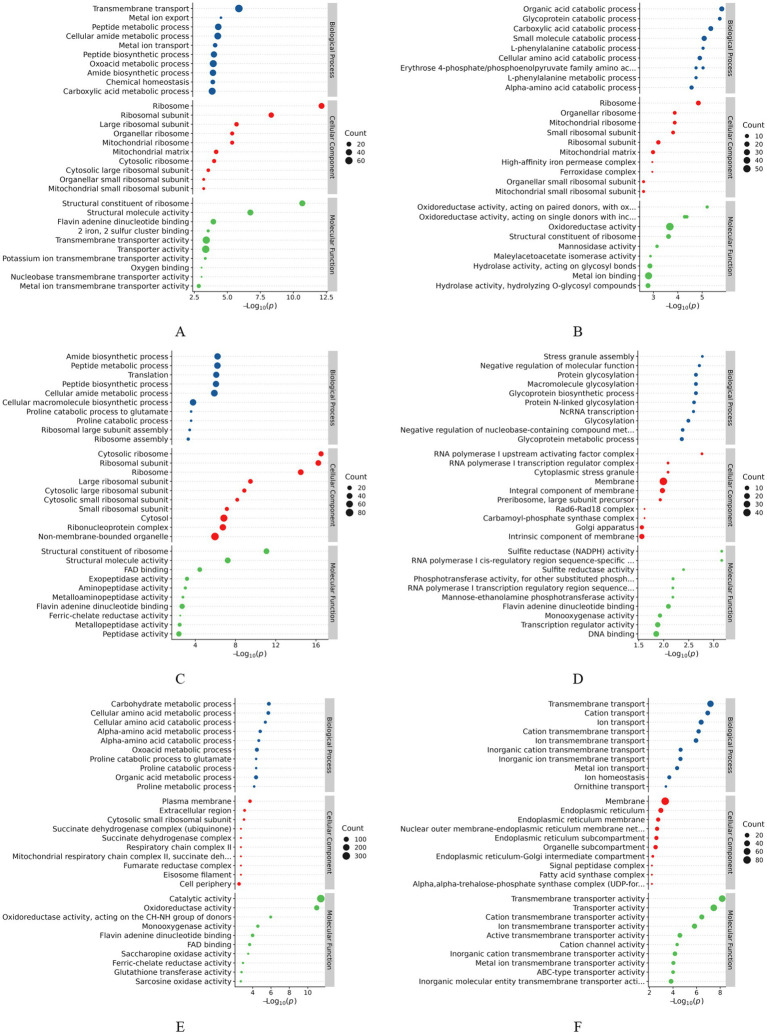
GO enrichment analysis of differentially expressed proteins. **(A)** A1 vs. B1; **(B)** A2 vs. B2; **(C)** A3 vs. B3; **(D)** A2 vs. A1; **(E)** A3 vs. A2; **(F)** A3 vs. A1.

The DEPs in the samples were analyzed for GO functions. These proteins belonged to three major categories: fungal molecular function (MF), biological process (BP), and CC. The top 10 GO terms with the smallest *p*-values, that is, the most significantly enriched terms, in each GO category were selected for comparison with the control group, and the results are shown in Figure 4. The results of the GO enrichment analysis (Figure 4A) of the DEPs in the 24-h samples (groups A1 and B1) revealed that the significantly enriched MF terms were structural constituent of the ribosome (GO:0003735), transmembrane transporter activity (GO:0022857), and transporter activity (GO:0005215); the significantly enriched BP terms were transmembrane transport (GO:0055085), metal ion export (GO:0070839), and peptide metabolic process (GO:0006518); and the significantly enriched CC terms were ribosome (GO:0005840), ribosomal subunit (GO:0044391), and large ribosomal subunit (GO:0015934). Thus, GO enrichment analysis of the DEPs in the early invasion stage revealed that these proteins were mainly associated with pathways related to ribosomal structure, transmembrane transport, and metabolic processes, suggesting that the fungus regulates protein synthesis, material transport, and metabolic processes in the host to achieve invasion and parasitism. In the analysis of the DEPs from the 48-h samples (groups A2 and B2) (Figure 4B), the significantly enriched MF terms were oxidoreductase activity (GO:0016491), structural constituent of ribosome (GO:0003735), hydrolase activity, and acting on glycosyl bonds (GO:0016798); these pathways could potentially affect the physiological state of the host by regulating redox reactions, ribosome function, and glycogen metabolism of the host. The significantly enriched BP terms were organic acid catabolic process (GO:0016054), glycerol catabolic process (GO:0016054), and glycogen catabolic process (GO:0016054), glycoprotein catabolic process (GO:0006516), carboxylic acid catabolic process (GO:0046395), and other pathways; these pathways may affect the host’s energy metabolism and material cycle by regulating the catabolic process of organic acids, glycoproteins, and carboxylic acids in the host. Energy metabolism and material cycling can help further catabolize the host. The significantly enriched CC terms were ribosome (GO:0005840), organellar ribosome (GO:0000313), and mitochondrial ribosome (GO:0005761); these pathways regulated the protein synthesis process and further enhanced ribosome-related functions. For DEPs (Figure 4C) in the 96-h samples (groups A3 and B3), the significantly enriched MF terms were structural constituent of ribosome (GO:0003735), structural molecule activity (GO:0005198), and FAD binding (GO:0071949); this finding implied a significant increase in the proteins binding to flavin adenine dinucleotide (FAD) in the treatment group, which may be involved in redox reactions or energy metabolism. The significantly enriched BP terms were amide biosynthetic process (GO:0043604), peptide metabolic process (GO:0006518), and translation (GO:0006412); amide and peptide metabolism as well as protein translation activities increased, suggesting that the metabolic function of the fungus is enhanced at the late stage of infestation. The significantly enriched CC terms were cytosolic ribosome (GO:0022626), ribosomal subunit (GO:0044391), and ribosome (GO:0005840). Thus, similar to the findings for the pre-infestation and mid-infestation phases, ribosome-associated cellular components were significantly enriched, indicating that the mycelium produces a stress response and that protein synthesis activities are increased after stimulation by the eggs during fungal infestation of the eggs.

Analysis of the DEPs between groups A1 and A2 (Figure 4D) showed that the significantly enriched MF terms were RNA polymerase I cis-regulatory region sequence-specific DNA binding (GO:0001165), sulfite reductase (NADPH) activity (GO:0004783), sulfite reductase activity (GO:0016002), and other pathways; this finding implied that changes in sulfite reductase activity may be related to the cellular demand for sulfur metabolism or stress response. The significantly enriched CC terms were RNA polymerase I upstream activating factor complex (GO:0000500), cytoplasmic stress granule (GO:0010494), and membrane (GO:0016020), which are involved in transmembrane transport, signaling, or regulation of membrane proteins. The significantly enriched BP terms were stress granule assembly (GO:0034063), negative regulation of molecular function (GO:0044092), and protein glycosylation (GO:0006486), implying that biological processes related to stress granule formation were significantly enhanced. Thus, the enrichment analysis of DEPs in the pre- and mid-infestation groups indicated that the fungus might be in a state of stress and that the increase in ribosome-related functions might be related to the regulation of protein synthesis, whereas the formation of stress granules and changes in membrane-associated proteins reflect the response of the mycelia to environmental changes. The analysis of DEPs between groups A2 and A3 (Figure 4E) showed that the significantly enriched CC terms were the plasma membrane (GO:0005886), extracellular region (GO:0005576), and cytosolic small ribosomal subunit (GO:0022627) and other pathways, along with a significant increase in proteins associated with the plasma membrane during the middle to late stages of infestation, suggesting that cell signaling, substance transport, or cell–cell interactions may be involved in the late stages of infestation. The significantly enriched MF terms were catalytic activity (GO:0003824), oxidoreductase activity (GO:0016491), oxidoreductase activity, acting on the CH–NH group of donors (GO:0016645), and other pathways, suggesting that the activity of enzymes related to redox reactions was significantly increased, which may be involved in energy metabolism or antioxidant reactions. The significantly enriched BP terms were carbohydrate metabolic process (GO:0005975), cellular amino acid metabolic process (GO:0006520), and alpha–amino acid metabolic process (GO:1901605). DEPs may affect energy supply and protein synthesis by regulating carbohydrate and amino acid metabolism. The analysis of DEPs between groups A1 and A3 (Figure 4F) showed that the significantly enriched CC terms were membrane (GO:0016020), endoplasmic reticulum (GO:0005783), and endoplasmic reticulum membrane (GO:0005789) as well as the nuclear outer membrane–endoplasmic reticulum membrane network (GO:0042175); the significantly enriched MF terms were transmembrane transporter activity (GO:0022857), transporter activity (GO:0005215), and cation transmembrane transporter activity (GO:0008324) terms; and the significantly enriched BP terms were transmembrane transport (GO:0055085), cation transport (GO:0006812), and ion transport (GO:0006811).

GO analysis of the functions of the DEPs revealed that the mycelia triggered a series of signal-transduction pathways under the stimulation of inducers. The number of fungal transporter activity-related proteins varied significantly after induction, and a variety of transmembrane proteins may be involved in processes such as host cell recognition, adhesion, and the formation of invasive structures. In the BP category, proteins with differential abundance were mainly involved in metabolic processes, cellular processes, bioregulation, and responses to stimuli. In terms of MF, DEPs were mainly associated with catalytic activity, binding, structural molecular activity, and translocation activity. Subcellular localization mainly occurred in the cytoplasm, nuclear membrane, and mitochondria. Secreted hydrolases, including proteases and chitinases, play a fundamental role in the degradation of eggshell components.

### KEGG analysis of differential proteins

**Figure 5:**
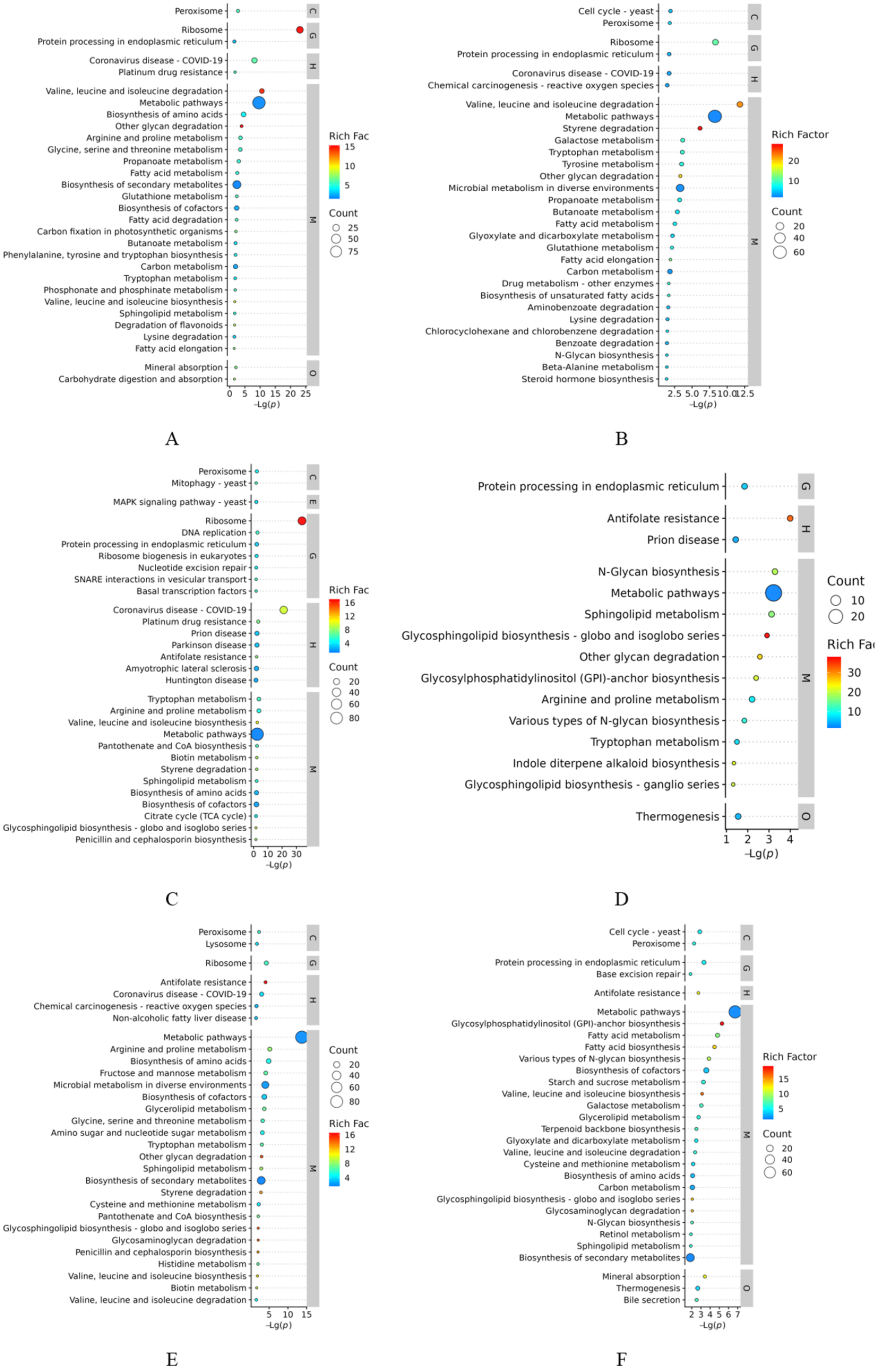
KEGG enrichment analysis of differentially expressed proteins. **(A)** A1 vs. B1; **(B)** A2 vs. B2; **(C)** A3 vs. B3; **(D)** A2 vs. A1; **(E)** A3 vs. A2; **(F)** p A3 vs. A1.

To determine the biological pathways corresponding to the three different infestation stages of *P. chlamydosporia*, these proteins were further mapped to the corresponding pathways in the KEGG database. The results of the KEGG enrichment analysis of the DEGs are shown in Figure 4. DEGs (Figure 5A) were annotated to 221 pathways in the comparative analysis of the 24-h treatment groups (A1 and B1), and 11 significantly enriched pathways were identified. These genes were related to ribosome; valine, leucine, and isoleucine degradation; metabolic pathways; biosynthesis of amino acids; other glycan degradation; arginine and proline metabolism; glycine, serine, and threonine metabolism; propanoate metabolism; and peroxisomes. KEGG analysis revealed significant enrichment of DEPs in metabolic pathways during the early stages of infestation, suggesting that the fungus was stimulated by inducers and that it enhanced the metabolism of exogenous substances, adapted to the host environment, and initiated the invasion mechanism. DEGs (Figure 5B) were annotated to 157 pathways in the comparative analysis of the 48-h treatment groups (A2 and B2), revealing 12 significantly enriched pathways. These pathways were valine, leucine, and isoleucine degradation; ribosomes; metabolic pathways; styrene degradation; galactose metabolism; tryptophan metabolism; tyrosine metabolism; other glycan degradation; microbial metabolism in diverse environments; propanoate metabolism; butanoate metabolism; and fatty acid metabolism. The enrichment of mycelial metabolic pathways in the middle infestation stage suggests that the fungus may adapt to the host environment by regulating various metabolic processes during infestation, and by degrading host glycosides to obtain energy and carbon sources, while destroying the host cell wall or extracellular matrix. DEGs (Figure 5C) were annotated to 256 pathways in the comparative analysis of the 96-h treatment groups (A3 and B3), and 45 significantly enriched pathways were identified. These genes were related to ribosome; tryptophan metabolism; arginine and proline metabolism; DNA replication; valine, leucine, and isoleucine biosynthesis; metabolic pathways; pantothenate and coenzyme A (CoA) biosynthesis; and peroxisome and biotin metabolism, including metabolic and cellular processes. In comparison with the pre- and intermediate-acting processes, the fine-tuned regulation of metabolic pathways and cell proliferation was enhanced.

**Figure 6:**
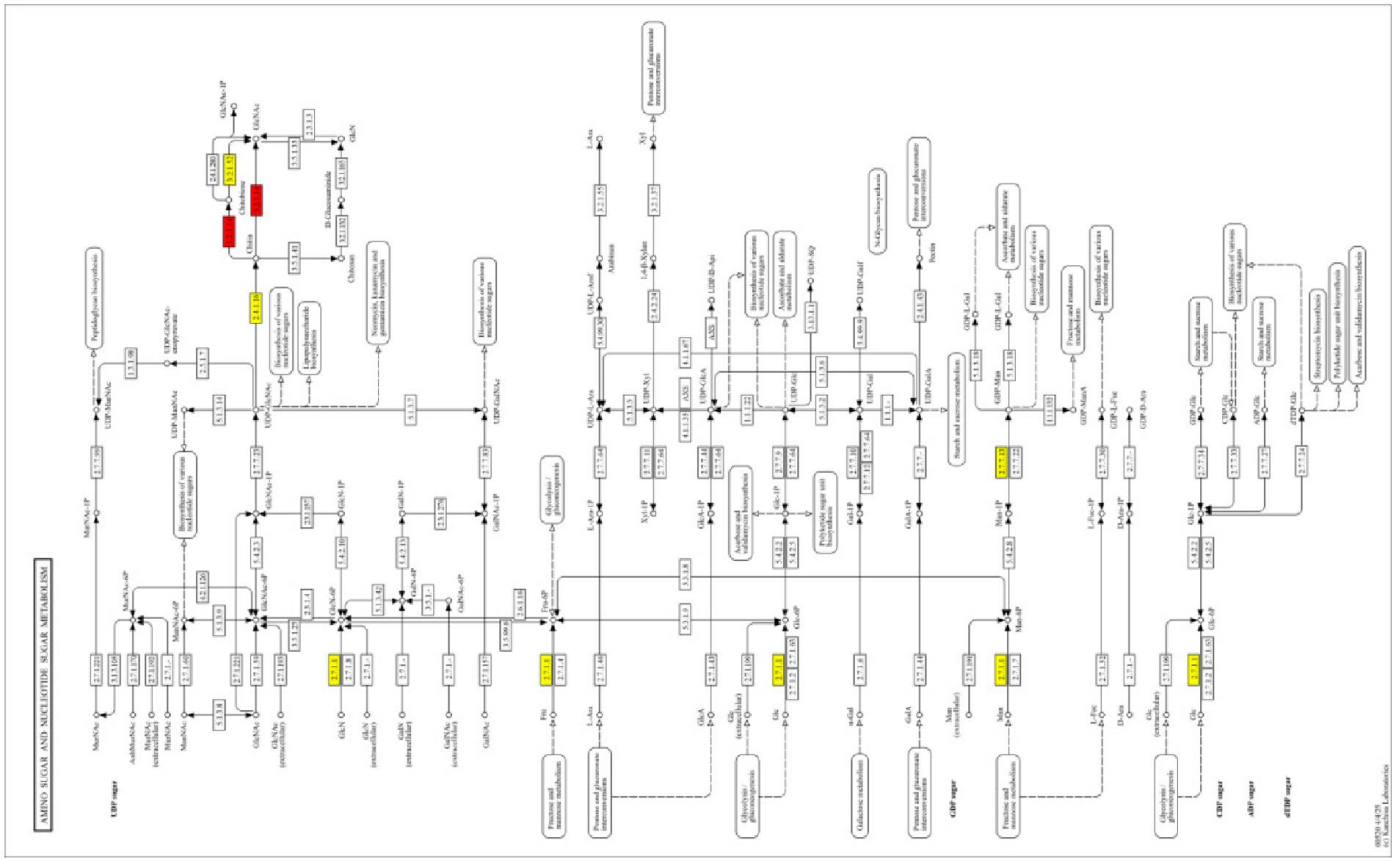
KEGG pathway amino sugar and nucleotide sugar metabolism (map00520). Red indicates significantly upregulated pathways.

DEGs (Figure 5D) were annotated to 111 pathways in the comparative analysis of the 24- and 48-h treatment groups (A1 vs. A2), resulting in 13 significantly enriched pathways. These genes were involved in *N*-glycan biosynthesis, metabolic pathways, sphingolipid metabolism, glycosphingolipid biosynthesis-globo and isoglobo series, other glycan degradation, glycosylphosphatidylinositol (GPI)-anchor biosynthesis, arginine and proline metabolism, protein processing in the endoplasmic reticulum, various types of *N*-glycan biosynthesis, thermogenesis, tryptophan metabolism, indole diterpene alkaloid biosynthesis, and glycosphingolipid biosynthesis-ganglio series. These findings suggest that early- to mid-infestation mycelia regulate *N*-glycan and GPI anchor biosynthesis, affect protein function and localization, and influence cell membrane structure and signaling by regulating sphingolipid and glycosphingolipid biosynthesis. DEGs (Figure 5E) were annotated to 208 pathways in the comparative analysis of the 48- and 96-h treatment groups (A2 vs. A3), and 38 significantly enriched pathways were identified. These genes were related to the following metabolic pathways: arginine and proline metabolism; biosynthesis of amino acids; ribosome, fructose, and mannose metabolism; microbial metabolism in diverse environments; biosynthesis of cofactors; glycerolipid metabolism; glycine, serine, and threonine metabolism; and amino sugar and nucleotide sugar metabolism. Nucleotide sugar metabolism and mid- to late-infestation development of mycelia involve the degradation and synthesis of amino acids to obtain nutrients, support their own growth, and regulate carbohydrate and lipid metabolism to increase the energy supply. DEGs (Figure 5F) were annotated to 203 pathways in the comparison of the 24- and 96-h treatment groups (A1 vs. A3), and 40 significantly enriched pathways were identified. These genes are related to metabolic pathways such as glycosylphosphatidylinositol (GPI)-anchor biosynthesis; fatty acid metabolism; fatty acid biosynthesis; various types of *N*-glycan biosynthesis; cofactor biosynthesis; mineral absorption; protein processing in the endoplasmic reticulum; starch and sucrose metabolism; and valine, leucine and isoleucine biosynthesis, where the endoplasmic reticulum is an important organelle for protein folding and processing. Enrichment of this pathway suggests that the fungus supports the process of infestation by regulating protein processing in the endoplasmic reticulum and affecting protein function and stability. Multiple stages showed significant enrichment of the chitin degradation pathway in amino sugar and nucleotide sugar metabolism, in which chitinase is mainly involved in the degradation of chitin (chitin) in the metabolic pathway. The degradation product of chitin (composed of *N*-acetylglucosamine and GlcNAc), GlcNAc, enters this pathway and is further metabolized to UDP-GlcNAc (used for glycosylation, peptidoglycan synthesis, and other processes), indicating that the chitinase-mediated amino sugar metabolism pathway is capable of degrading chitin in worm eggs, as shown in Figure 6.

### Results of fluorescence quantitative PCR analyses

To validate the findings of proteomic sequencing, eight DEPs. qRT-PCR analysis of the corresponding genes was performed using the cDNAs obtained at 24, 48, and 96 h during the action of *P. chlamydosporia* on the eggs as templates to validate the expression of the target genes. The validation results revealed that the trends in the gene expression profiles obtained by qRT-PCR were positively correlated with the RNA-seq data.

## Discussion

To understand the mechanism of action of *P. chlamydosporia* in infesting insect eggs, this study, on the basis of previous research on the insecticidal effect of the fungus, conducted a proteomic analysis of the fungus to establish a histological database of *P. chlamydosporia* ordinary nutrient hyphae and hyphae containing infestation structures. The key genes and major protein species of the egg-parasitic fungus that kill insect eggs and their differential expression were determined by histological techniques.

Proteomic analysis revealed that DEPs in the pre-infestation phase of fungal eggs were enriched mainly in the structural constituents of ribosomes, transmembrane transport, and peptide metabolic process pathways. Among them, the levels of the MFS transporter and the adhesin protein Mad1 were significantly increased (Qi and Peng, 2019). Peteira et al. (2015) used complementary DNA (cDNA)-amplified fragment length polymorphism (AFLP) based on transcriptional profiling to identify genes involved in the pathogenicity of nematode eggs infested with *Puccinia thickettsii* and reported that genes encoding transcription factors, transporter proteins, and enzymes involved in the metabolism of the fungus were enriched (Peteira et al., 2015). This is consistent with the enrichment trends of transporters and metabolism-related pathways in the present study. Mycelia in the early stages of infestation require rapid adaptation to the host environment to initiate infestation. By enhancing metabolic pathways and protein synthesis, the fungus can rapidly acquire nutrients and synthesize proteins required for infestation, and by enhancing the antioxidant response, the fungus can respond to the oxidative stress generated by the host to ensure its own survival and infestation, which helps the fungus cope with the host’s defense response (Qi et al., 2022). Among these pathways, the MAPK signaling pathway mediates fungal immune evasion and promotes infection by upregulating transcriptional repressors and histidine phosphotransferases, while ABC transporter-mediated lipid transport is crucial for membrane formation of structures such as appressorium (Wawra et al., 2016). These findings suggest that the fungus supports the infestation process by increasing fungal protein synthesis and material transport capacity to rapidly establish the metabolic base required for infestation and to support the infestation process by regulating the translation mechanism of the fungus to promote the synthesis of its own proteins (Andersson et al., 2014).

In the middle stage of infestation (48 h), the functional focus of the DEPs shifted to oxidoreductase activity, hydrolase activity, glycosyl bonding, organic acid catabolic processes, and glycoprotein catabolic processes. These changes suggest that at this stage, the fungus is beginning to utilize host energy storage substances (e.g., carbohydrates and organic acids) and regulate the oxidative stress state of the host through redox reactions to maintain its growth advantage (Kotze, 2003). In addition, enrichment of the mitochondrial ribosome further suggests that the fungus may weaken the defenses of eggs by interfering with the energy metabolism (e.g., ATP synthesis) of the host. Fungi need to adapt to the host environment and acquire more nutrients, and their metabolic pathways diversify to obtain more nutrients to support their own growth and reproduction. At this stage, an increase in glycoside degradation and lipid metabolism can help the fungus destroy the host cell wall or extracellular matrix. Nematode eggshells consist of an outer yolk layer composed of proteins, a middle layer of chitin (composed of a protein matrix embedded in chitin microfibrils), and an inner lipid layer (Wrońska et al., 2023; Bird and McClure, 1976; Clarke et al., 1967; Atkins et al., 2003). Thus, the simultaneous production of proteases (P32, VCP1, SCP1) (Ward et al., 2012; Lopez-Llorca et al., 2010; Olivares-Bernabeu and Lopez-Llorca, 2002), chitinases (Yang et al., 2007; Wang et al., 2005; Thalita et al., 2017; Clavero-Camacho et al., 2024), and lipases is essential for egg penetration and colonization by *P. chlamydosporia* (Ramesh et al., 2016; Huang et al., 2004). The fungus destroys the host cell wall or extracellular matrix by degrading host glycosides and lipids, resulting in damage to the host cell structure and further weakening the host’s defense ability; simultaneously, the fungus degrades amino acids, glycans, and lipids through hydrolytic enzymes and lipases to obtain nutrients and support its own growth and predation. Moreover, the fungus degrades host amino acids, sugars, and lipids using hydrolytic enzymes to obtain nutrients and support its growth, robbing the host of its nutrient sources and leading to host cell failure (Elliott et al., 2019; Fry et al., 2018).

By the late stage of infestation (96 h), the DEPs were significantly enriched in pathways such as FAD binding, amide biosynthetic process, and translation, indicating a progressive increase in fungal metabolic activity, which may have involved more complex redox reactions and the regulation of protein synthesis. The persistent enrichment of ribosome-related pathways (e.g., cytosolic ribosomes) suggests that protein synthesis remains a key process in the late stage of infestation, whereas the enrichment of the endoplasmic reticulum and transmembrane transporter activity may reflect the modification of the host cell membrane system by the fungus to promote nutrient uptake or toxicant secretion. Fungi have to maintain their growth and reproduction while responding to host defense. Fungi can support proliferation and infestation by fine-tuning their metabolic pathways and enhancing DNA replication (Yang et al., 2013). Sustained enhancement of the antioxidant response helps the fungus cope with oxidative stress in the host and ensures successful completion of the infestation process. Throughout the course of infestation, the fungus affects the function and stability of host proteins and interferes with the normal physiological processes of the host by regulating protein glycosylation and processing. The fungus gradually adapts to the host environment and completes infestation through dynamic regulation of metabolic pathways and cellular functions. From the early to late stages of infestation, fungi utilize various mechanisms, such as nutrient deprivation, destruction of cell structure, oxidative stress, and protein function interference, which ultimately lead to egg death.

Comparative analyses of the findings obtained during different infestation periods revealed that the fungus underwent stress adaptation during infestation. For example, the enrichment of sulfite reductase activity and stress granule assembly suggests that the fungus has to cope with sulfur metabolic stress or oxidative stress in the host, whereas the enrichment of the plasma membrane and oxidoreductase activity in the later stages may involve intercellular signaling and antioxidant defenses to maintain a long-term parasitic state. Together, these pathways provide the necessary material and functional basis for the fungal infestation of worm eggs at multiple levels, including regulation of gene expression and protein synthesis. Stimulation of mycelia by worm eggs triggers a series of signaling pathways that contribute to the survival, propagation, and further infestation of the fungus within the worm eggs.

Our findings showing a significant increase in hydrolase activity between 24 and 48 h, coupled with broader proteome changes, indicated substantial transcriptional reprogramming triggered by nutrient stress as the fungus transitioned from a saprophytic to a parasitic lifestyle. This suggests the involvement of specific gene families, particularly hydrolases and transport proteins, potentially facilitating the endosymbiotic behavior crucial for parasitism (Larriba et al., 2014). Although the pronounced upregulation of hydrolases at 48 h aligns with expectations for host penetration and nutrient acquisition, the extent of the increase across multiple enzyme classes within this narrow timeframe is particularly noteworthy and underscores the intensity of this metabolic shift. These findings provide a crucial molecular timeline of the adaptive response of *P. chlamydosporia*, which enable it to counter host defenses while sustaining its own growth and reproduction. Our results align with, yet highlight distinctions from, previous findings outlining the mechanisms employed by other nematophagous fungi. For instance, while urea acts as a key environmental signal triggering the saprophytic-to-parasitic switch in *Arthrobotrys oligospora*, and *Hirsutella minnesotensis* employs diverse signaling pathways to parasitize various nematode hosts, the response by *P. chlamydosporia* appears to be heavily reliant on the rapid, coordinated induction of hydrolytic enzymes and transport functions as detected in its proteome. This mechanistic diversity underscores the evolutionary flexibility of fungal parasitism strategies. Understanding these specific molecular adaptations in *P. chlamydosporia,* a promising biocontrol agent against nematodes, is vital for the development of effective and targeted biological control strategies.

However, this study focused exclusively on the fungal proteome, which is a major limitation since it does not capture the concurrent host responses or the dynamic molecular dialogue occurring at the host-pathogen interface during infestation. Future studies should prioritize integrated proteomic and transcriptomic analyses of both fungi and their host nematodes during infection to characterize these critical interactions. Furthermore, targeted functional studies of the identified hydrolases and transport proteins, potentially through gene knockout or RNAi approaches, are essential to confirm their specific roles in virulence and adaptation, ultimately providing strategies for enhancing the biocontrol efficacy of *P. chlamydosporia*.

## Conclusion

Proteomic analysis showed that *P. chlamydosporia* adapts to the host environment and completes its invasion by dynamically regulating gene expression and metabolic networks. RNA-binding proteins and ribosome biosynthesis-related genes were upregulated in the early stage of invasion (24 h), which allowed the fungus to respond to the environment and rapidly initiate the invasive program, enhancing transporter function and the antioxidant response to address the oxidative stress of the host. In the middle stage of infestation (48 h), oxidoreductase and glycoside/lipid metabolism pathways were activated to destroy the host cell wall and remove nutrients, and serine proteases (e.g., VCP1 and P32) and ghrelinase (GH18 family) acted synergistically to degrade the eggshell protein layer and ghrelin barrier. In comparison with the pre- and mid-term stages, the late stage of infestation (96 h) enhanced the fine-tuned regulation of metabolic pathways and cell proliferation. Overall, the enhanced functions of metabolic pathways and cellular processes at the transcriptional and translational levels during the infestation process indicate complex metabolic regulation of the fungus during egg infestation, which involves energy acquisition, protein synthesis and modification, and the response to oxidative stress.

## Data Availability

The mass spectrometry proteomics data generated during this study have been deposited to the Massive repository (https://massive.ucsd.edu) with the dataset identifier MSV000098741.
