# Supplementary figures and images for: Proteomic analyses of the regulatory mechanisms underlying Pochonia chlamydosporia infection in Parascaris equorum eggs
Source: Front Microbiol. 2025 Oct 15;16:1644912. doi: 10.3389/fmicb.2025.1644912 (PMC12569784; doi:10.3389/fmicb.2025.1644912)

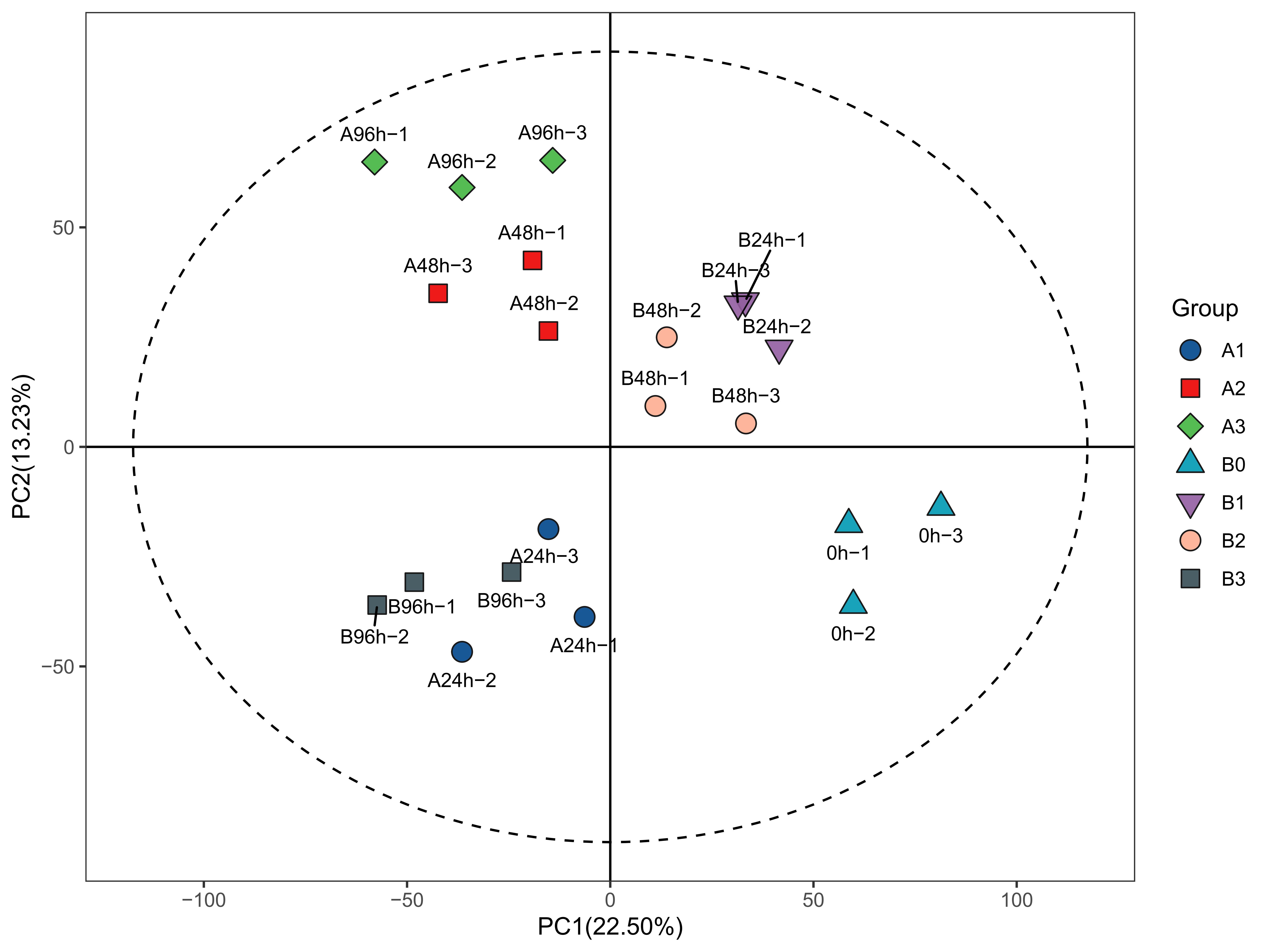

Supplement: Supplementary file 1 [file Image_1.JPEG]
